# Temporal trends in mortality due to coronary heart disease in Germany from 1998 to 2023

**DOI:** 10.25646/13178

**Published:** 2025-06-11

**Authors:** Henriette Steppuhn, Jens Baumert, Viktoria Rücker, Kai Günther, Annelene Wengler, Fabian Tetzlaff, Hannelore Neuhauser

**Affiliations:** 1 Robert Koch Institute, Department for Epidemiology and Health Monitoring, Berlin, Germany; 2 Institut for Clinical Epidemiology und Biometry, University of Würzburg, Germany; 3 DZHK (German Centre for Cardiovascular Research)

**Keywords:** Mortality, Coronary heart disease, CHD, Acute myocardial infarction, Chronic CHD, Time series analysis, Joinpoint regression

## Abstract

**Background:**

Coronary heart disease (CHD) is the leading cause of death in Germany. Comprehensive analyses of long-term trends in CHD mortality that also distinguish between acute myocardial infarction (AMI) and non-AMI-related chronic CHD are currently lacking.

**Method:**

Age-specific and age-standardised CHD mortality rates for the period 1998 – 2023 were calculated based on data from the cause-of-death statistics of the Federal Statistical Office of Germany. Annual percentage changes (APC) and average annual percentage changes (AAPC) were estimated using joinpoint regression analysis.

**Results:**

Between 1998 and 2023, the average annual change in age-standardised CHD mortality rates for women was -3.9 % ((-4.1) – (-3.7)) per year, compared with -3.2 % ((-3.3) – (-3.0)) for men. However, since the 2010s, the downward trend in CHD mortality has flattened, particularly among those aged 60 to 74 years. In the analysis by ICD subgroups, mortality rates for chronic CHD declined less sharply than for AMI over the entire period 1998 – 2023, especially among men.

**Conclusions:**

The flattening of the CHD mortality trend, particularly among middle-aged adults over the last decade, and the smaller decline in chronic CHD mortality, especially among men, require further exploration in order to identify unmet needs at various levels of prevention for specific life stages. In addition, the influence of the COVID-19 pandemic on CHD mortality trends should be further investigated.

## 1. Introduction

Coronary heart disease (CHD) is characterized by narrowing or occlusion of the coronary arteries that supply blood to the heart muscle. The chronic form of CHD is distinguished from acute myocardial infarction (AMI) as a life-threatening acute event that can lead to permanent damage to the heart muscle tissue [1]. Heart failure is a major complication of CHD that also contributes to CHD mortality [2, 3]. CHD is the leading cause of death in Germany [4, 5] and CHD mortality rates in Germany are above the European average [6, 7]. In burden of disease (mortality and morbidity) estimations for CHD compared to other chronic diseases, mortality contributes much stronger to the losses of life years than health impairments [8].

Recently, different trends in morbidity and 28-day case fatality rates for AMI by age and sex have been observed. Analyses of myocardial infarction registry data from the Augsburg region between 2009 and 2015 showed a decline in AMI incidence and 28-day case fatality rates among adults aged 25 to 84 [9]. However, this decline was only consistent among the elderly (aged 75 – 84). In contrast, among those under 75 years of age, a stagnation in AMI incidence and 28-day case fatality rates was observed between 2004 and 2015, particularly in younger age groups [9]. Current analyses also suggest that there was excess mortality due to AMI during the pandemic years [10, 11]. In addition to the direct acute and post-acute consequences of SARS-CoV-2 infections [12, 13], indirect effects of the COVID-19 pandemic could have also contributed as well, including changes in the continuity of care and effects on patient behaviour, such as changes in lifestyle or healthcare seeking behaviour [11, 14, 15]. However, there are no recent studies on temporal trends in mortality due to chronic CHD. The aim of the present analysis was therefore to examine changes in long-term trends in CHD mortality between 1998 and 2023. We focussed our analysis on sex- and age-specific developments and further distinguished between ICD-10 subgroups for AMI and (non-AMI-related) chronic CHD.

## 2. Methods

### 2.1 Data sources

The official statistics on causes of death include all deaths of deceased persons with permanent residence in Germany and contain information on the frequency of diseases or external events leading to death (underlying causes of death) [16]. The cause of death is documented on the death certificate by a doctor after death has been confirmed and a post-mortem examination has been carried out. In addition to the underlying condition (which caused death), previous, subsequent, and concomitant conditions that contributed to the death are also recorded. The underlying cause of death is registered by the federal state statistical offices in accordance with the WHO coding guidelines [17–20]. In further electronic processing of the data, the information on the underlying condition is compiled in the official cause-of-death statistics of the Federal Statistical Office of Germany (Statistisches Bundesamt, Destatis) of Germany and annual numbers on causes of deaths are aggregated by sex and age group and provided on the online database of federal health reporting (Gesundheitsberichterstattung, GBE) (www.gbe-bund.de) [21, 22]. However, additional information on previous, subsequent, or concomitant diseases on the death certificate is not yet recorded or made available on a nationwide basis.

For our analyses, we used sex- and age-specific mortality data coded according to the 10th revision of the International Statistical Classification of Diseases and Related Health Problems (ICD-10) for the following causes: CHD (ICD-10: I20 – 25), AMI (ICD-10: I21 – 22) and chronic CHD (ICD-10: I25). The corresponding figures for the annual average population numbers were based on projections from census data collected in 1987 in the former Federal Republic of Germany (FRG) and in 1990 in the former German Democratic Republic (GDR) for the years 1998 to 2010 [23]. From 2011 onwards, projections from the 2011 census were used [24].

**Key messages**

► Coronary heart disease (CHD) was the most common cause of death in Germany in 2023, with 119,800 deaths.
► The age-standardised mortality rate for CHD was lower for women than for men throughout the entire period from 1998 to 2023.
► Between 1998 and 2023, age-standardised mortality rates for CHD declined more sharply among women (3.9 % per year) than among men (3.2 %).
► Since the 2010s, the downward trend in CHD mortality has flattened, particularly among women and men aged 60 to 74.
► Regular collection of reliable data on temporal trends in cardiovascular risk factors is crucial to identify unmet needs in cardiovascular disease prevention.

### 2.2 Statistical methods

Missing values for cells with fewer than three cases were set to zero. In the result section, the raw number of deaths was reported rounded to the nearest hundred. Sex-specific crude mortality rates were calculated by dividing the annual number of deaths by the respective annual average population per 100,000 inhabitants for each 5-year age group. The resulting mortality rates were directly age-standardised based on the 2013 European standard population as the reference population [25]. A log-linear regression model was used for trend analysis and annual percentage changes in age-standardised mortality rates were determined [26, 27]. We applied a joinpoint regression model in order to identify years in which statistically significant trend changes, known as joinpoints, occurred [26]. At a joinpoint, the decline in a mortality may accelerate or slow down, but stagnation or a trend reversal may also occur. Joinpoints thus divide the time series into periods. Annual percentage changes (APC) intervals are estimated for each of these periods. In our analyses, the maximum number of joinpoints was set at four, as only a limited number of joinpoints (*n* = 4 for ≤ 26 observation years) allow meaningful estimates for a given number of observations [28].

In addition, the average annual percentage change (AAPC) was calculated for the entire observation period 1998 – 2023 as the weighted mean of all APCs taking the respective length of the individual APC intervals (duration in years) into account. Based on the AAPC, the average APCs could thus be described with a single number over a period of several years [29]. The joinpoint regression analyses were performed using the Joinpoint Regression Program, version 5.0.2.0 (Statistical Research and Applications Branch, National Cancer Institute) [26, 30]. All other statistical analyses were carried out using the statistical software STATA, version 17 (StataCorp [31]). In addition to analysing age-specific rates in 5-year age groups (< 5, 5 – 9, 10 – 14, …, 80 – 84, 85 – 89, ≥ 90 years), age-standardised analyses were also performed for the three age groups < 60 years, 60 – 74 years, and ≥ 75 years.

## 3. Results

### 3.1 Temporal development of CHD mortality

**Figure 1:**
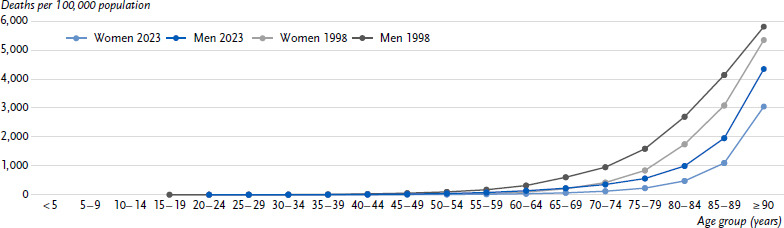
Age-specific crude mortality rates for CHD (ICD-10: I20 – 25) in Germany in 1998 and 2023 by sex. Source: Cause-of-Death Statistics, Federal Statistical Office of Germany CHD = Coronary heart disease ICD-10: International Statistical Classification of Diseases and Related Health Problems, 10th Revision [32]

**Figure 2:**
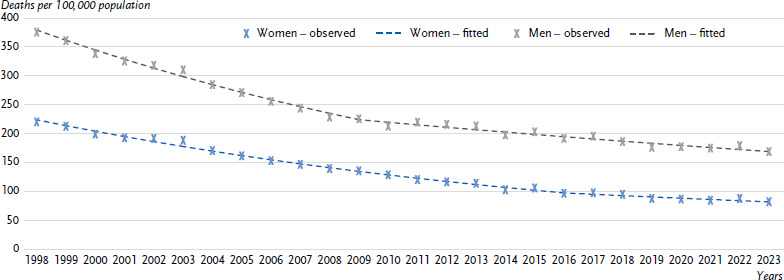
Observed and fitted age-standardised mortality rates for CHD (ICD-10: I20 – 25) in Germany from 1998 to 2023 by sex. Source: Cause-of-Death Statistics, Federal Statistical Office of Germany CHD = Coronary heart disease ICD-10: International Statistical Classification of Diseases and Related Health Problems, 10th Revision [32]

Overall, fewer women (51,200) than men (68,600) were reported to have died due to CHD in 2023. The crude mortality rate was also lower for women than for men (119.4 vs. 164.7 per 100,000 inhabitants) and increased with age for both sexes (Figure 1). Sex differences in CHD mortality rates remained even after considering differences in age structure. Between 1998 and 2023, age-standardised CHD mortality rates fell more sharply among women (62.8 %) than among men (55.1 %, Figure 2).

Figure 2 shows the temporal development of annual age-standardised CHD mortality rates between 1998 and 2023. Over the entire period from 1998 to 2023, CHD mortality rates among women fell by an average of 3.9 % per year, which was a greater decline than among men (-3.2 % per year, Table 1). In both sexes, only one joinpoint was identified at which there was a significant change in trend. This joinpoint was recorded for women in 2016 and for men in 2009 and marked a flattening of downward trends in both sexes. Among women, the decline in CHD mortality rates slowed from -4.5 % per year (1998 – 2016) to -2.4 % per year (2016 – 2023), while among men, it flattened from -4.7 % per year (1998 – 2009) to -2.0 % per year (2009 – 2023; Table 1). Most recently, starting in 2016, there has been no significant downward trend among women.

### 3.2 Age-specific analyses of the temporal development of CHD mortality

**Annex Figure 1:**
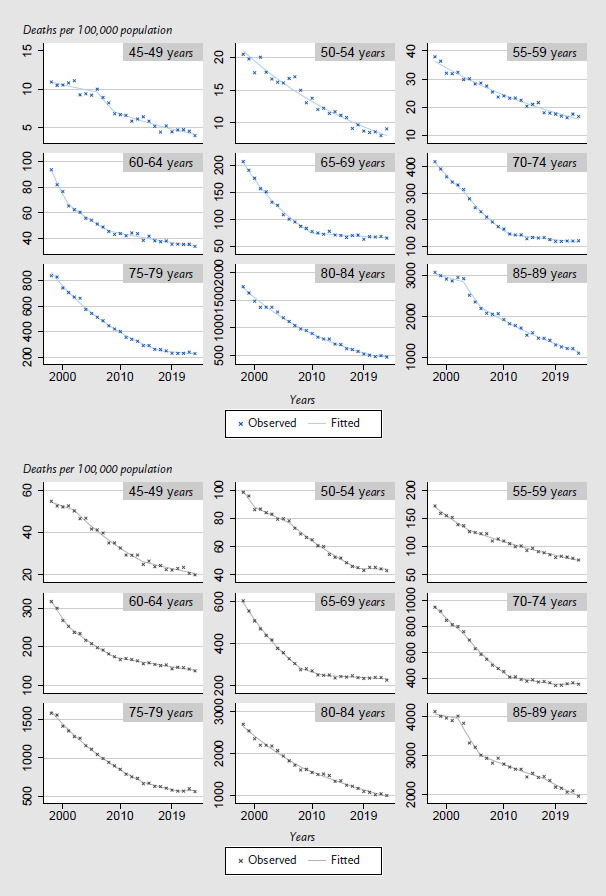
Observed and fitted crude CHD mortality rates (ICD-10: I20 – 25) for women (blue, upper figure) and men (grey, lower figure) by 5-year age groups (45–89 years) from 1998 to 2023 in Germany. Source: Cause-of-death statistics, Federal statistical office of Germany CHD = Coronary heart disease ICD-10: International Statistical Classification of Diseases and Related Health Problems, 10th Revision [32]

Table 2 and Annex Figure 1 show the results of the analyses by age group. The slowdown in downward trends in CHD mortality during the 2010s was most pronounced among women and men in the 60 to 74 age group (Annex Figure 1). In this age group, a joinpoint was identified for both sexes in 2011 (Table 2), and the annual percentage decline in CHD mortality rates was lower in the period 2011–2023 (women: -1.6 %, men: -1.1 %) than in the period 1998 – 2011 (women: -7.5 %, men -6.2 %, Table 2). From 2008 onwards, the downward trend among men over 75 years of age also flattened from -4.2 % to -2.1 % (Table 2). From 2016 onwards, however, no relevant downward trend was observed among women over 75 years, nor among men under 60 years from 2018 onwards (Table 2).

### 3.3 Temporal development of mortality for AMI and chronic CHD

**Figure 3:**
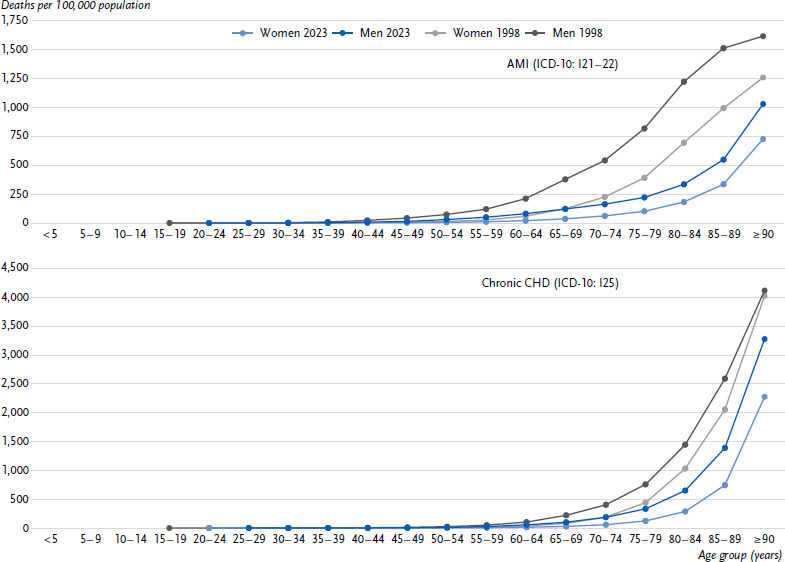
Age-specific crude mortality rates for AMI (ICD-10: I21 – 22, upper figure) and chronic CHD (ICD-10: I25, lower figure) in Germany in 1998 and 2023 by sex. Source: Cause-of-Death Statistics, Federal Statistical Office of Germany AMI = Acute myocardial infarction, CHD = Coronary heart disease ICD-10: International Statistical Classification of Diseases and Related Health Problems, 10th Revision [32]

In 2023, fewer women than men were reported to have died due to AMI (17,400 vs. 26,400) or chronic CHD (33,000 vs. 41,400). The crude mortality rates for AMI were also lower in women (40.7 per 100,000 inhabitants) than in men (63.4 per 100,000 inhabitants) and increased with age (Figure 3). For chronic CHD, similar sex differences (77.0 in women vs. 99.5 per 100,000 inhabitants in men) and age gradients were observed (Figure 3). The sex differences remained even after considering the different age structures in both groups. Between 1998 and 2023, age-standardised AMI mortality rates fell by about two-thirds (66.5 % vs. 65.4 %) for women and men, and thus more sharply than for chronic CHD (60.7 % vs. 45.4 %).

**Figure 4:**
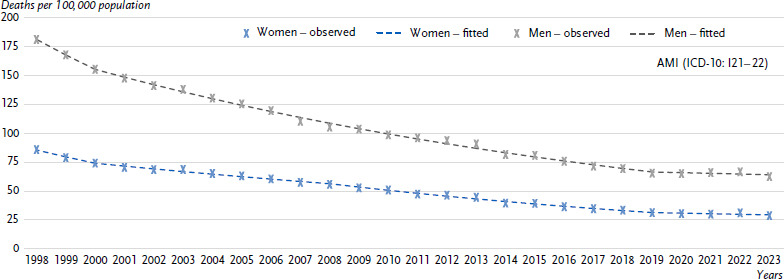

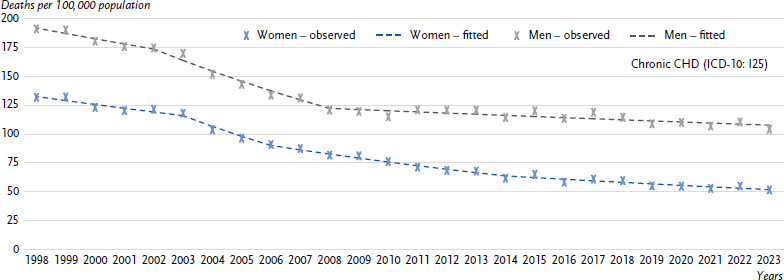
Observed and fitted age-standardised mortality rates for AMI (ICD-10: I21 – 22, upper figure) and chronic CHD (ICD-10: I25, lower figure) 1998 – 2023 in Germany by sex. Source: Cause-of-Death Statistics, Federal Statistical Office of Germany AMI = Acute myocardial infarction, CHD = Coronary heart disease ICD-10: International Statistical Classification of Diseases and Related Health Problems, 10th Revision [32]

Figure 4 shows the development of age-standardised annual mortality rates for AMI and chronic CHD over time. Over the entire observation period from 1998 to 2023, AMI mortality rates fell by an average of 4.2 % per year for women and 4.1 % per year for men. For chronic CHD, however, mortality rates declined more sharply between 1998 and 2023 for women (-3.7 % per year) than for men (-2.3 % per year). Most recently, no relevant change in mortality for AMI (from 2019 onwards) and chronic CHD (from 2014 onwards for women and from 2008 onwards for men) could be observed (Annex Table 1).

## 4. Discussion

Our study provides an insight into the long-term development of CHD mortality in Germany. We were able to show that age-standardised CHD mortality rates fell by 3.9 % per year between 1998 and 2023 among women, which was a stronger decline than among men, where the rate fell by 3.2 % per year. However, this decline flattened significantly in the last decade, particularly among adults aged 60 to 74 in both sexes. In the analysis by ICD subgroups, mortality rates for chronic CHD declined less than for AMI over the entire period from 1998 to 2023. This difference was smaller for women than for men.

During the observation period, starting at the end of the 1990s, a significant decline in CHD mortality was likewise observed in many western industrialized countries [33]. A recent meta-analysis quantified the impact of changes in the prevalence of risk factors compared to changes in the medical therapy on temporal trends in CHD mortality. It was found that positive developments in the reduction of risk factors such as smoking had a stronger effect on reducing CHD mortality than improvements in the treatment of CHD patients [33]. So far, no such studies have been carried out in Germany. However, the stronger decline in mortality due to AMI compared to chronic CHD observed in the current analysis could indicate a significant influence of changes in acute care on CHD mortality trends. In mortality statistics, AMI is only registered as an underlying cause of death within a period of 28 days after an acute myocardial infarction. From day 29 onwards, however, a myocardial infarction (ICD-10: I25.2 old myocardial infarction) is coded as the underlying cause of death within the ICD-10 subgroup of chronic CHD (I25.8 other forms of chronic IHD), provided that no other cause qualifies as the underlying condition [34]. Improvements in management and reperfusion therapy of AMI patients may therefore have contributed to the decline in AMI mortality [9, 35–37]. In addition, certified specialised units for the treatment of acute chest pain (chest pain units) have been introduced [38, 39], which are particularly beneficial for patients without specific changes (ST segment elevation) in the electrocardiogram who have chest pain caused by CHD or other life-threatening conditions [3].

In addition to improvements in acute care, some findings in Germany suggest that positive changes in the development of risk factors have also been relevant. Based on the data available for the period up to 2015, there was a decrease in the incidence of AMI [9, 35, 36, 40] accompanied by a decline in the frequency of important CHD risk factors [41]. Analyses of nationwide surveys of adults in Germany showed that the average systolic blood pressure in the population decreased between the two survey waves in 1997 – 1999 (BGS98) and 2008 – 2011 (DEGS1). Moreover, the analyses indicated that there was an improvement in hypertension control among people with high blood pressure [42–44]. During the same period, a decrease in total cholesterol and triglyceride levels measured in blood serum and an increase in the use of lipid-lowering drugs in the population were also observed [41, 45]. In addition, the frequency of current and heavy smoking as well as physical inactivity declined between the two survey waves [41, 46].

Further improvements were reported with regard to the frequency of recurrent heart attacks and the long-term survival of heart attack patients [9, 40]. At the same time, there were indications of positive developments in pharmacological secondary prevention, which can affect the course of CHD in certain patients and reduce the risk of death. For example, the use of antiplatelet agents (24.0 % vs. 59.6 %), beta-receptor blockers (24.7 % vs. 65.5 %), agents with an effect on the renin-angiotensin system (31.6 % vs. 69.0 %) and statins (18.5 % vs. 56.2 %) increased among patients with a self-reported lifetime diagnosis of CHD between 1997 – 1999 and 2008 – 2011 [47]. Based on data from patients enrolled in a disease management program (DMP) for CHD, there was also an improvement over time in the target low-density lipoprotein (LDL) cholesterol levels and a significant increase in the prescription of CHD-related drugs, especially in patients with concomitant heart failure [48–50]. The DMP for CHD was introduced in 2003 to reduce cardiovascular morbidity and mortality and had 1.9 million participants in 2023 [51].

A second important finding of our analysis is that the downward trend in CHD mortality has slowed significantly over the last decade. Overall, the decline in CHD mortality has also flattened in many other western industrialised countries. This development has been linked to an increase in the prevalence of risk factors such as obesity and diabetes [52, 53]. For Germany, recent studies based on nationwide health surveys between 2003 and 2023 showed a continuous rise in the prevalence of obesity [54]. The overall prevalence of smoking declined slightly, but recently stagnated at an above-average level compared to the EU average, particularly with regard to the prevalence of daily smoking [46, 54–56]. Moreover, the nationwide surveys of adults in Germany in 1997 – 1999 (BGS98) and 2008 – 2011 (DEGS1), showed a stagnation in the prevalence of type 2 diabetes after considering blood tests (HbA1c levels) [57]. The latest available data from the nationwide survey of adults in Germany between 2008 to 2011 (DEGS1) further indicated that, despite overall positive developments, there were still potential deficits in the early detection and treatment of high blood pressure and lipid metabolism disorders in the population [43, 44, 58]. Current evaluations of data from the DMP for CHD and clinical myocardial infarction registry data also describe potential for improvement in terms of smoking cessation and the achievement of target blood pressure and cholesterol levels among myocardial infarction and CHD patients [49, 60, 61]. Our age-specific analysis showed that downward trends in CHD mortality has flattened in the last decade, particularly among adults aged 60 to 74 years. These results are consistent with a study based on myocardial infarction registry data from the Augsburg region, which observed different trends in morbidity and 28-day case fatality rates of AMI in the elderly compared to younger age groups. Between 2009 and 2015, there was a decline in the rates of first and recurrent AMI and in AMI case fatality among adults aged 75 to 84 [9]. In contrast, among those under 75 years of age, AMI and case fatality rates stagnated between 2004 and 2015, with this trend being particularly noticeable in younger age groups [9].

Our analyses according to ICD subgroups also showed that the observed downward trend in AMI mortality was no longer noticeable from 2019 onwards. This stagnating trend among women and men until 2023 may be related to the results of studies that identified excess mortality due to AMI [10, 11]. Acute and post-acute consequences of SARS-CoV-2 infections were discussed as possible causes of this excess [12, 13]. In addition to these direct effects, indirect consequences of the pandemic might also be relevant. These include, in particular, changes in the continuity of care for CHD patients and possible effects on the lifestyle or healthcare seeking behaviour [11, 14, 15]. For example, significant declines in hospitalisation rates for AMI were observed, particularly during the first lockdown periods [11, 14, 15]. In addition, the complex mechanisms between competing events, such as COVID-19 as a new cause of death and selection effects (harvesting effects) during period events such as the COVID-19 pandemic, can lead to considerable uncertainty in the interpretation of time series of underlying cause of death data [62, 63]. Further research and more comprehensive cause of death data (on the causal chain) are therefore urgently needed to better understand changes in mortality patterns over time.

The strength of our work lies in the analysis of long-term trends in CHD mortality using the official cause-of-death statistics including all deceased persons with permanent residence in Germany. We performed regression analyses in order to quantify relative changes within defined time intervals identified on the basis of joinpoint modeling. The magnitude of the relative changes in individual APCs could be compared between population groups. We highlight differences by sex and age as well as in the development of AMI and chronic CHD, adding to findings on the long-term development of non-AMI-related CHD mortality. Moreover, joinpoint regression is used for a hypothesis-free identification of points in time at which changes or joinpoints in mortality trends can be observed. Our study thus follows a hypothesis-generating approach that can serve as a starting point for further in-depth analyses.

However, it should be noted that our work is based on data from the official mortality statistics, which are subject to temporal changes in the coding of underlying causes of death according to WHO guidelines [64, 65]. Studies have indicated that the frequency of so-called ill-defined cardiovascular causes of death, which according to WHO guidelines should not be coded as underlying conditions, declined in Germany between 2000 and 2016 [65]. These ill-defined causes of death include heart failure which usually forms an intermediate link (intermediate cause of death) in the sequence of causes leading to death (causal chain) [3, 34]. However, heart failure is still frequently reported as the underlying cause of death in Germany although at least half of the cases can be attributed to CHD [65, 67]. In addition, the selection of age-related causes of death, which are essentially competing underlying conditions, might have changed over time [68]. This is of particular relevance for the analysis of mortality trends in the elderly.

To date, the official mortality statistics in Germany only record the underlying cause of death. No other information on the causal chain or on previous and concomitant diseases from the death certificate can be used for further sensitivity analyses. It is therefore not possible to investigate the extent to which there have been shifts in the classification of quasi-competing or ill-defined causes of death over time. This limitation is particularly relevant in the elderly, but does not fundamentally call into question the finding that the significant downward trend in mortality for both AMI and chronic CHD during the 2010s has flattened, especially among middle-aged adults. A greater decline in AMI morbidity and case fatality rates among people aged 75 and over compared to younger age groups was also observed based on population-based data of the Augsburg myocardial infraction registry [9]. Analyses of clinical registry data indicated improvements in acute care for older, more morbid AMI patients [37]. In addition, we observed a smaller decrease in mortality due to chronic CHD than in mortality due to AMI. In order to understand the causes for this development, reliable data on CHD risk factors are needed based on periodic population-representative health examination surveys over time. However, such data have not been collected since 2011. This is of particular relevance as current analyses indicate that spatial differences in AMI mortality are mainly due to differences in the prevalence of CHD risk factors [69]. Since changes in lifestyle factors such as diet or smoking can lead to a short-term decline in cardiovascular mortality, timely data on the temporal development of CVD risk factors are essential for understanding temporal trends in mortality [70–72].

In Germany, the decades-long decline in CHD mortality has flattened since the 2010s and has recently levelled off for specific sex, age, and ICD subgroups. This is highly relevant as CHD remains the leading cause of death in Germany and the burden of disease (mortality and morbidity) due to CHD is mainly attributable to CHD mortality [8]. For a reduction in CHD mortality a combination of population-wide prevention and control of risk factors as well as appropriate acute and long-term care for AMI and chronic CHD patients is needed. Informed decision making for both preventive and health care measures need regularly collected population-wide comprehensive data. These include data on risk factors and diseases from periodic nationwide and regionally comparable health examination surveys, data on healthcare utilization and medical care, as well as on cause-specific mortality. In this context, it is highly relevant to consider age-related and quasi-competing causes of death in the elderly, and focus mortality analyses on premature, preventable and treatable CHD mortality in the under-75 age group [73]. For nationwide mortality analyses, information on all diseases documented on the death certificates (causal chain) and socio-demographic data of the deceased should also be available. This would enable in-depth investigations of dynamic changes in mortality patterns, e.g., during a pandemic or future health crises.

## Figures and Tables

**Table 1:** Annual percentage change (APC) and average annual percentage change (AAPC) in age-standardised mortality rates for CHD (ICD-10: I20 – 25) from 1998 to 2023 by sex. Source: Cause-of-Death Statistics, Federal Statistical Office of Germany

**Women**		
**Period**	**APC, %**	**(95 % CI)**
1998 – 2016	-4,5	((-4,9) – (-4,3))
2016 – 2023	-2,4	((-3,4) – 0,3)
**Total period**	**AAPC, %**	**(95 % CI)**
1998 – 2023	-3,9	((-4,1) – (-3,7))
**Men**		
**Period**	**APC, %**	**(95 % CI)**
1998 – 2009	-4,7	((-5,2) – (-4,2))
2009 – 2023	-2,0	((-2,3) – (-1,6))
**Total period**	**AAPC, %**	**(95 % CI)**
1998 – 2023	-3,2	((-3,3) – (-3,0))

CHD = Coronary heart disease

ICD-10: International Statistical Classification of Diseases and Related Health Problems, 10th Revision [32]

**Table 2:** Annual percentage change (APC) and average annual percentage change (AAPC) in age-standardised mortality rates for CHD (ICD-10: I20 – 25) by sex and age group from 1998 to 2023. Source: Cause-of-Death Statistics, Federal Statistical Office of Germany

Age group	Women			Men		
**< 60 years**	**Period**	**APC, %**	**(95 % CI)**	**Period**	**APC, %**	**(95 % CI)**
	1998 – 2023	-3,7	(-4,0) – (-3,5))	1998 – 2018	-3,8	((-4,0) – (-3,7))
				2018 – 2023	-1,1	((-2,6) – 2,4)
	**Total period**	**AAPC, %**	**(95 % CI)**	**Total period**	**AAPC, %**	**(95 % CI)**
	1998 – 2023	-3,7	((-4,0) – (-3,5))	1998 – 2023	-3,3	((-3,5) – (-3,1))
**60 – 74 years**	**Period**	**APC, %**	**(95 % CI)**	**Period**	**APC, %**	**(95 % CI)**
	1998 – 2011	-7,5	((-7,9) – (-7,2))	1998 – 2011	-6,2	((-6,5) – (-5,9))
	2011 – 2023	-1,6	((-2,1) – (-1,0))	2011 – 2023	-1,1	((-1,4) – (-0,6))
	**Total period**	**AAPC, %**	**(95 % CI)**	**Total period**	**AAPC, %**	**(95 % CI)**
	1998 – 2023	-4,7	((-4,9) – (-4,6))	1998 – 2023	-3,7	((-3,9) – (-3,6))
**≥ 75 years**	**Period**	**APC, %**	**(95 % CI)**	**Period**	**APC, %**	**(95 % CI)**
	1998 – 2003	-3,0	((-4,1) – 0,2)	1998 – 2008	-4,2	((-5,0) – (-3,7))
	2003 – 2016	-4,5	((-7,3) – (-4,2))	2008 – 2023	-2,1	((-2,4) – (-1,7))
	2016 – 2023	-2,7	((-3,8) – 0,8)			
	**Total period**	**AAPC, %**	**(95 % CI)**	**Total period**	**AAPC, %**	**(95 % CI)**
	1998 – 2023	-3,7	((-4,0) – (-3,4))	1998 – 2023	-2,9	((-3,1) – (-2,8))

CHD = Coronary heart disease

ICD-10: International Statistical Classification of Diseases and Related Health Problems, 10th Revision [32]

**Annex Table 1:** Annual percentage change (APC) and average annual percentage change (AAPC) in age-standardised mortality rates due to AMI (I21 – 22) and chronic CHD (I25) by sex from 1998 to 2023. Source: Cause-of-death statistics, Federal Statistical Office of Germany

	Women			Men		
**AMI**	**Period**	**APC, %**	**(95 % CI)**	**Period**	**APC, %**	**(95 % CI)**
	1998 – 2000	-6,9	((-8,6) – (-4,1))	1998 – 2000	-7,4	((-8,9) - (-4,5))
	2000 – 2008	-3,4	((-4,0) – (-1,6))	2000 – 2019	-4,4	((-4,6) – (-4,0))
	2008 – 2019	-5,2	((-6,4) – (-4,8))	2019 – 2023	-0,9	((-2,5) – 2,4)
	2019 – 2023	-1,6	((-3,4) – 1,8)			
	**Total period**	**AAPC, %**	**(95 % CI)**	**Total period**	**AAPC, %**	**(95 % CI)**
	1998 – 2023	-4,2	((-4,4) – (-4,0))	1998 – 2023	-4,1	((-4,2) – (-3,8))
**Chronic CHD**	**Period**	**APC, %**	**(95 % CI)**	**Period**	**APC, %**	**(95 % CI)**
	1998 – 2003	-2,6	((-6,3) – 1,3)	1998 – 2002	-2,5	((-4,7) – 2,1)
	2003 – 2006	-8,0	((-9,7) – (-0,8))	2002 – 2008	-5,7	((-9,3) – 1,8)
	2006 – 2014	-4,2	((-7,6) – 0,5)	2008 – 2023	-0,8	((-2,0) – 0,3)
	2014 – 2023	-2,3	((7,3) – 1,7)			
	**Total period**	**AAPC, %**	**(95 % CI)**	**Total period**	**AAPC, %**	**(95 % CI)**
	1998 – 2023	-3,7	((-4,1) – (-3,3))	1998 – 2023	-2,3	((-2,6) – (-1,9))

AMI = Acute myocardial infarction, CHD = Coronary heart disease

ICD-10: International Statistical Classification of Diseases and Related Health Problems, 10th Revision [32]

## References

[1] Bundesärztekammer (BÄK), Kassenärztliche Bundesvereinigung (KBV), Arbeitsgemeinschaft der Wissenschaftlichen Medizinischen Fachgesellschaften (AWMF). Nationale VersorgungsLeitlinie Chronische KHK, Langfassung, Version 7.0. 2024. 2024. Available from: https://register.awmf.org/de/leitlinien/detail/nvl-004.

[2] Naghavi M Makela S Foreman K O‘Brien J Pourmalek F Lozano R. Algorithms for enhancing public health utility of national causes-of-death data. Popul Health Metr. 2010;8:9. Epub 20100510. doi: 10.1186/1478-7954-8-9. 20459720 10.1186/1478-7954-8-9 PMC2873308

[3] Deutsche Herzstiftung e. V. Deutscher Herzbericht – Update 2024. Frankfurt am Main; 2024.

[4] Wengler A Rommel A Plass D Gruhl H Leddin J Ziese T. Years of Life Lost to Death – A Comprehensive Analysis of Mortality in Germany Conducted as Part of the BURDEN 2020 Project. Dtsch Arztebl Int. 2021;118(9):137-44. doi: 10.3238/arztebl.m2021.0148. 33958031 10.3238/arztebl.m2021.0148 PMC8212398

[5] Tetzlaff F Sauerberg M Grigoriev P Tetzlaff J Muhlichen M Baumert J. Age-specific and cause-specific mortality contributions to the socioeconomic gap in life expectancy in Germany, 2003-21: an ecological study. Lancet Public Health. 2024;9(5):e295-e305. doi: 10.1016/S2468-2667(24)00049-5. 38702094 10.1016/S2468-2667(24)00049-5

[6] Causes of death – standardised death rate by NUTS 2 region of residence [Internet]. 2024. Available from: https://ec.europa.eu/eurostat/databrowser/view/hlth_cd_asdr2$defaultview/default/table?lang=en.

[7] Organisation for Economic Collaboration and Development (OECD)/ European Union. Health at a Glance: Europe 2022: State of Health in the EU Cycle, OECD Publishing, Paris. 2022. doi: 10.1787/507433b0-en.

[8] Porst M Lippe EV Leddin J Anton A Wengler A Breitkreuz J. The Burden of Disease in Germany at the National and Regional Level. Dtsch Arztebl Int. 2022;119(46):785-92. doi: 10.3238/arztebl.m2022.0314. 36350160 10.3238/arztebl.m2022.0314 PMC9902892

[9] Kramer C Meisinger C Kirchberger I Heier M Kuch B Thilo C. Epidemiological trends in mortality, event rates and case fatality of acute myocardial infarction from 2004 to 2015: results from the KORA MI registry. Ann Med. 2021;53(1):2142-52. doi: 10.1080/07853890.2021.2002926. 34779325 10.1080/07853890.2021.2002926 PMC8604473

[10] Baumert J Scheidt-Nave C Steppuhn H Tetzlaff F Kraywinkel K An der Heiden M. Altered Mortality From Selected Non-communicable Diseases During the COVID-19 Pandemic in Germany in 2020 and 2021. Dtsch Arztebl Int. 2024;121(4):135-6. doi: 10.3238/arztebl. m2023.0262. 38518297 10.3238/arztebl.m2023.0262 PMC11019757

[11] Han L Zhao S Li S Gu S Deng X Yang L. Excess cardiovascular mortality across multiple COVID-19 waves in the United States from March 2020 to March 2022. Nat Cardiovasc Res. 2023;2(3):322-33. Epub 20230227. doi: 10.1038/s44161-023-00220-2. 39195997 10.1038/s44161-023-00220-2

[12] Tsampasian V Back M Bernardi M Cavarretta E Debski M Gati S. Cardiovascular disease as part of Long COVID: A systematic review. Eur J Prev Cardiol. 2024. Epub 20240221. doi: 10.1093/eurjpc/zwae070. 10.1093/eurjpc/zwae070 38381595

[13] Eberhardt N Noval MG Kaur R Amadori L Gildea M Sajja S. SARS-CoV-2 infection triggers pro-atherogenic inflammatory responses in human coronary vessels. Nat Cardiovasc Res. 2023;2(10):899-916. Epub 20230928. doi: 10.1038/s44161-023-00336-5. 38076343 10.1038/s44161-023-00336-5 PMC10702930

[14] Drogan D Gerloff C Scholz KH Günster C. Die stationäre Behandlung von Patientinnen und Patienten mit Herzinfarkt und Schlaganfall während der Covid-19-Pandemie. 2022. In: Krankenhaus-Report 2022 – Patientenversorgung während der Pandemie. 10.1007/978-3-662-64685-4.

[15] Scheidt-Nave C Fehr A Haller S Sarganas G Steppuhn H Truthmann J. Providing actionable evidence in Public Health – The 2018 international workshop on evidence-based public health at the Robert Koch Institute, Berlin. J Health Monit. 2020;5(Suppl 3):3-6. Epub 20200604. doi: 10.25646/6499. 10.25646/6499 PMC8734086 35146283

[16] Statistisches Bundesamt (Destatis). Statistik der Sterbefälle. Qualitätsbericht zur Statistik der Sterbefälle 2022-2023. 2024. Available from: https://www.destatis.de/DE/Methoden/Qualitaet/Qualitaets-berichte/Bevoelkerung/sterbefaelle.pdf?__blob=publicationFile.

[17] World Health Organisation (WHO). ICD-10 WHO Version. 2016. Available from: https://klassifikationen.bfarm.de/icd-10-gm/kode-suche/htmlgm2024/index.htm.

[18] Buschner A Grunwald-Muhlberger A. [Influence of methodological changes on unicausal cause-of-death statistics and potentials of a multicausal data basis]. Bundesgesundheitsbl. 2019;62(12):1476-84. doi: 10.1007/s00103-019-03048-z. 10.1007/s00103-019-03048-z 31720737

[19] Gleich S Viehover S Teipel A Drubba S Turlik V Hirl B. [Death certificates – an underestimated source of information for statistics, judicature, public health, and science]. Bundesgesundheitsbl. 2019;62(12): 1415-21. doi: 10.1007/s00103-019-03042-5. 10.1007/s00103-019-03042-5 31686152

[20] Eckert O Vogel U. [Cause-of-death statistics and ICD, quo vadis?]. Bundesgesundheitsbl. 2018;61(7):796-805. doi: 10.1007/s00103-018-2756-5. 10.1007/s00103-018-2756-5 29869705

[21] Statistisches Bundesamt. Grundlagen der Todesursachenstatistik. Available from: https://www.destatis.de/DE/Themen/Gesellschaft-Umwelt/Gesundheit/Todesursachen/Tabellen/todesursachen.pdf?__blob=publicationFile.

[22] Gesundheitsberichterstattung (GBE). Sterbefälle, Sterbeziffern (je 100.000 Einwohner, altersstandardisiert) (ab 1998). Gliederungsmerkmale: Jahre, Region, Alter, Geschlecht, Nationalität, ICD-10, Art der Standardisierung. [Available from: https://www.gbe-bund.de.

[23] Gesundheitsberichterstattung (GBE). Bevölkerung im Jahresdurchschnitt. Gliederungsmerkmale: Jahre, Region, Alter, Geschlecht, Nationalität (Grundlage Zensus BRD. 1987, DDR 1990). [Available from: https://www.gbe-bund.de.

[24] Gesundheitsberichterstattung (GBE). Bevölkerung im Jahresdurchschnitt. Gliederungsmerkmale: Jahre, Region, Alter, Geschlecht, Nationalität (Grundlage Zensus 2011). Available from: https://www.gbe-bund.de.

[25] Eurostat. Revision of the European Standard Population. Report of Eurostat’s task force – 2013 edition. Luxemburg; 2013.

[26] Kim HJ Fay MP Feuer EJ Midthune DN. Permutation tests for join-point regression with applications to cancer rates. Stat Med. 2000;19(3):335-51. doi: 10.1002/(sici)1097-0258(20000215)19:3<335::aid-sim336>3.0.co;2-z. 10649300 10.1002/(sici)1097-0258(20000215)19:3<335::aid-sim336>3.0.co;2-z

[27] Statistical Methodology and Applications Branch Surveillance Research Program National Cancer Institute. Joinpoint Regression Program, Version 5.0.2.0.

[28] National Cancer Institute. Number of Joinpoints. Available from: https://surveillance.cancer.gov/help/joinpoint/setting-parameters/method-and-parameters-tab/number-of-joinpoints.

[29] National Cancer Institute. Average Annual Percent Change (AAPC) and Confidence Interval. 2024. Available from: https://surveillance.cancer.gov/help/joinpoint/tech-help/frequently-asked-questions/aapc-definition.

[30] Statistical Research and Applications Branch of the National Cancer Institute. Software „Joinpoint Regression Programm“, Version 4.6.0.0. April, 2018.

[31] StataCorp. Stata Statistical Software: Release 17. College Station, TX: StataCorp LLC. 2021.

[32] Bundesinstitut für Arzneimittel und Medizinprodukte (BfArM). ICD-10-WHO Version 2019. 2019. Available from: https://klassifikationen.bfarm.de/icd-10-who/kode-suche/htmlamtl2019/index.htm.

[33] Ahmadi M Lanphear B. The impact of clinical and population strategies on coronary heart disease mortality: an assessment of Rose‘s big idea. BMC Public Health. 2022;22(1):14. Epub 20220106. doi: 10.1186/s12889-021-12421-0. 34991551 10.1186/s12889-021-12421-0 PMC8734316

[34] Deutsches Institut für Medizinische Dokumentation und Information (DIMDI). Internationale statistische Klassifikation der Krankheiten und verwandter Gesundheitsprobleme 10. Revision – WHO-Ausgabe – Version 2019. Stand: August 2018. Band 2 – Regelwerk.

[35] Neumann JT Gossling A Sorensen NA Blankenberg S Magnussen C Westermann D. Temporal trends in incidence and outcome of acute coronary syndrome. Clin Res Cardiol. 2020;109(9):1186-92. Epub 20200207. doi: 10.1007/s00392-020-01612-1. 32034482 10.1007/s00392-020-01612-1

[36] Degano IR Salomaa V Veronesi G Ferrieres J Kirchberger I Laks T. Twenty-five-year trends in myocardial infarction attack and mortality rates, and case-fatality, in six European populations. Heart. 2015;101(17):1413-21. Epub 20150408. doi: 10.1136/heartjnl-2014-307310. 25855798 10.1136/heartjnl-2014-307310

[37] Bestehorn K Bestehorn M Fleck E D`Ancona G Ince H Eggebrecht H. Increase of in-Hospital Mortality of Stemi-Patients After PCI? A Risk Adjusted Analysis of German Quality Assurance Data 2008-2013. Journal of Cardiology & Cardiovascular Therapy. 2019;13(3):1-5. doi:10.19080/JOCCT.2019.13.555865.

[38] Deutsche Gesellschaft für Kardiologie – Herz- und Kreislaufforschung e. V. (DGK). Zertifizierte CPUs, Stand 24.10.2024. 2024. Available from: https://cpu.dgk.org/zertifizierte-cpus/.

[39] Breuckmann F Rassaf T Hochadel M Giannitsis E Munzel T Senges J. German chest pain unit registry: data review after the first decade of certification. Herz. 2021;46(Suppl 1):24-32. Epub 20200330. doi: 10.1007/s00059-020-04912-4. 32232516 10.1007/s00059-020-04912-4

[40] Tetzlaff J Tetzlaff F Geyer S Sperlich S Epping J. Widening or narrowing income inequalities in myocardial infarction? Time trends in life years free of myocardial infarction and after incidence. Popul Health Metr. 2021;19(1):47. Epub 20211224. doi: 10.1186/s12963-021-00280-1. 34952590 10.1186/s12963-021-00280-1 PMC8709953

[41] Finger JD Busch MA Du Y Heidemann C Knopf H Kuhnert R. Time Trends in Cardiometabolic Risk Factors in Adults. Dtsch Arztebl Int. 2016;113(42):712-9. doi: 10.3238/arztebl.2016.0712. 27866566 10.3238/arztebl.2016.0712 PMC5143790

[42] Neuhauser H Diederichs C Boeing H Felix SB Junger C Lorbeer R. Hypertension in Germany. Dtsch Arztebl Int. 2016;113(48):809-15. doi: 10.3238/arztebl.2016.0809. 28073425 10.3238/arztebl.2016.0809 PMC5241792

[43] Sarganas G Knopf H Grams D Neuhauser HK. Trends in Antihypertensive Medication Use and Blood Pressure Control Among Adults With Hypertension in Germany. Am J Hypertens. 2016;29(1):104-13. Epub 20150511. doi: 10.1093/ajh/hpv067. 25968124 10.1093/ajh/hpv067

[44] Neuhauser HK Adler C Rosario AS Diederichs C Ellert U. Hypertension prevalence, awareness, treatment and control in Germany 1998 and 2008-11. J Hum Hypertens. 2015;29(4):247-53. Epub 20141002. doi: 10.1038/jhh.2014.82. 25273858 10.1038/jhh.2014.82

[45] Truthmann J Schienkiewitz A Busch MA Mensink GB Du Y Bosy-Westphal A. Changes in mean serum lipids among adults in Germany: results from National Health Surveys 1997-99 and 2008-11. BMC Public Health. 2016;16:240. Epub 20160308. doi: 10.1186/s12889-016-2826-2. 26956524 10.1186/s12889-016-2826-2 PMC4784325

[46] Zeiher J Finger JD Kuntz B Hoebel J Lampert T Starker A. [Trends in smoking among adults in Germany: Evidence from seven population-based health surveys from 1991-2015]. Bundesgesundheitsbl. 2018;61(11):1365-76. doi: 10.1007/s00103-018-2817-9. 10.1007/s00103-018-2817-9 30215104

[47] Knopf H Busch MA Du Y Grams D Scheidt-Nave C Sarganas G. [Secondary prevention of coronary heart disease in women and men in Germany from 1997-1999 and from 2008-2011 – Trend analysis with two national health population surveys]. Bundesgesundheitsbl. 2019;62(7):861-9. doi: 10.1007/s00103-019-02975-1. 10.1007/s00103-019-02975-1 31187183

[48] Berendes A Potthoff F. Bericht der strukturierten Behandlungsprogramme der gesetzlichen Krankenkassen – Indikation Koronare Herzkrankheit (KHK), Erstellt durch Medivcal Netcare GmbH (MNC) und infas. Münster; 2022. Available from: https://www.g-ba.de/down-loads/17-98-5325/2020-06-15_DMP-KHK_Evaluationsbericht.pdf.

[49] Groos S Kretschmann J Weber A Hagen B. Qualitätsbericht 2022. Disease-Management-Programme in Nordrhein. Düsseldorf; 2022. Available from: https://www.kvno.de/fileadmin/shared/pdf/online/quali/KVNO_DMP_Qualitaetsbericht_2022.pdf.

[50] Groos S Kretschmann J Macare C Weber A Hagen B. Qualitätsbericht 2019. Disease-Management-Programme in Nordrhein. Düsseldorf; 2019. Available from: https://www.kvno.de/fileadmin/shared/pdf/print/berichte/dmp-berichte/qualbe_dmp19.pdf.

[51] Kassenärztliche Bundesvereinigung (KBV). Entwicklung der Anzahl eingeschriebener Patientinnen und Patienten im DMP KHK über den Zeitraum 2007 bis 2023. 2024. Available from: https://www.kbv.de/media/sp/DMP_KHK_Patienten.pdf.

[52] Organisation for Economic Collaboration and Development (OECD). Is Cardiovascular Disease Slowing Improvements in Life Expectancy? OECD and The King‘s Fund Workshop Proceedings 2020. 2020. Available from: https://www.oecd.org/health/is-cardiovascular-disease-slowing-improvements-in-life-expectancy-47a04a11-en.htm. doi: 10.1787/47a04a11 -en.

[53] Emmert-Fees KMF Luhar S O‘Flaherty M Kypridemos C Laxy M. Forecasting the mortality burden of coronary heart disease and stroke in Germany: National trends and regional inequalities. Int J Cardiol. 2023;393:131359. Epub 20230926. doi: 10.1016/j.ijcard.2023.131359. 37757987 10.1016/j.ijcard.2023.131359

[54] Starker A Schienkiewitz A Damerow S Kuhnert R. Prevalence of obesity and smoking among adults in Germany – trends from 2003 to 2023. J Health Monit. 2025;10(1):e 13038. doi: 10.25646/13038. 10.25646/13038 PMC11973604 40196747

[55] Daily smokers of cigarettes by sex age and income quintile [Internet]. 2022. Available from: https://ec.europa.eu/eurostat/databrowser/view/hlth_ehis_sk3i/default/table?lang=en.

[56] Smoking of tobacco products by sex age and educational attainment level [Internet]. 2022. Available from: https://ec.europa.eu/eurostat/databrowser/view/hlth_ehis_sk1e/default/table?lang=en.

[57] Heidemann C Du Y Paprott R Haftenberger M Rathmann W Scheidt-Nave C. Temporal changes in the prevalence of diagnosed diabetes, undiagnosed diabetes and prediabetes: findings from the German Health Interview and Examination Surveys in 1997-1999 and 2008-2011. Diabet Med. 2016;33(10):1406-14. Epub 20151123. doi: 10.1111/dme.13008. 26498983 10.1111/dme.13008

[58] Scheidt-Nave C Du Y Knopf H Schienkiewitz A Ziese T Nowossadeck E. [Prevalence of dyslipidemia among adults in Germany: results of the German Health Interview and Examination Survey for Adults (DEGS 1)]. Bundesgesundheitsbl. 2013;56(5-6):661-7. doi: 10.1007/s00103-013-1670-0. 10.1007/s00103-013-1670-0 23703484

[59] Baldus S Lauterbach K. Prevention-centered health care in Germany – a nation in need to turn the tide. Eur J Epidemiol. 2023;38(8):835-7. Epub 20230731. doi: 10.1007/s10654-023-01030-3. 37524897 10.1007/s10654-023-01030-3 PMC10421807

[60] Zeymer U Goss F Werdan K. GULLIVE-R – Guideline adherence and risk assessment after acute myocardial infarction in real life in Germany – a quality improvement and awareness registry of the German Cardiac Society. 2023. Available from: https://dgk.org/daten/2022-04-dgk-gullive-r.pdf.

[61] Kassenärztliche Bundesvereinigung (KBV). Disease-Management-Programm Koronare Herzkrankheit – Qualitätszielerreichung 2022. 2022. Available from: https://www.kbv.de/media/sp/DMP_KHK_Ergebnisse_QS.pdf.

[62] Luy M Di Giulio P Di Lego V Lazarevic P Sauerberg M. Life Expectancy: Frequently Used, but Hardly Understood. Gerontology. 2020;66(1):95-104. Epub 20190807. doi: 10.1159/000500955. 31390630 10.1159/000500955 PMC7026938

[63] Luy M Sauerberg M Muszyńska-Spielauer M. Decrease in Life Expectancy in Germany in 2020: Men from Eastern Germany Most Affected. Comparative Population Studies. 2021;41. doi: 10.12765/CPoS-2021-20.

[64] Stolpe S Kowall B Stang A. The Quality of Cause-Of-Death Statistics After the Introduction of the Electronic Coding System Iris/Muse-an Analysis of Mortality Data, 2005-2019. Dtsch Arztebl Int. 2023;120(46):793-4. doi: 10.3238/arztebl.m2023.0190. 38099602 10.3238/arztebl.m2023.0190 PMC10762840

[65] Stolpe S Stang A. [Noninformative coding of causes of death in cardiovascular deaths: effects on the mortality rate for ischemic heart disease]. Bundesgesundheitsbl. 2019;62(12):1458-67. doi: 10.1007/s00103-019-03050-5. 10.1007/s00103-019-03050-5 31720736

[66] Wengler A Rommel A Plass D Gruhl H Leddin J Porst M. [ICD coding of causes of death: challenges for calculating the burden of disease in Germany]. Bundesgesundheitsbl. 2019;62(12):1485-92. doi: 10.1007/s00103-019-03054-1. 10.1007/s00103-019-03054-1 31758220

[67] Stork S Handrock R Jacob J Walker J Calado F Lahoz R. Epidemiology of heart failure in Germany: a retrospective database study. Clin Res Cardiol. 2017;106(11):913-22. Epub 20170726. doi: 10.1007/s00392-017-1137-7. 28748265 10.1007/s00392-017-1137-7 PMC5655572

[68] Stolpe S Kowall B Stang A. Decline of coronary heart disease mortality is strongly effected by changing patterns of underlying causes of death: an analysis of mortality data from 27 countries of the WHO European region 2000 and 2013. Eur J Epidemiol. 2021;36(1):57-68. Epub 20201128. doi: 10.1007/s10654-020-00699-0. 33247420 10.1007/s10654-020-00699-0 PMC7847455

[69] Ebeling M Muhlichen M Talback M Rau R Goedel A Klusener S. Disease incidence and not case fatality drives the rural disadvantage in myocardial-infarction-related mortality in Germany. Prev Med. 2024;179:107833. Epub 20231223. doi: 10.1016/j.ypmed.2023.107833. 38145875 10.1016/j.ypmed.2023.107833

[70] Capewell S O‘Flaherty M. Can dietary changes rapidly decrease cardiovascular mortality rates? Eur Heart J. 2011;32(10):1187-9. Epub 20110302. doi: 10.1093/eurheartj/ehr049. 21367835 10.1093/eurheartj/ehr049

[71] Gao M Li Y Wang F Zhang S Qu Z Wan X. The effect of smoke-free legislation on the mortality rate of acute myocardial infarction: a meta-analysis. BMC Public Health. 2019;19(1):1269. Epub 20190918. doi: 10.1186/s12889-019-7408-7. 31533693 10.1186/s12889-019-7408-7 PMC6749716

[72] Frazer K Callinan JE McHugh J van Baarsel S Clarke A Doherty K. Legislative smoking bans for reducing harms from secondhand smoke exposure, smoking prevalence and tobacco consumption. Cochrane Database Syst Rev. 2016;2(2):CD005992. Epub 20160204. doi: 10.1002/14651858.CD005992.pub3. 26842828 10.1002/14651858.CD005992.pub3 PMC6486282

[73] Weber A Reisig V Buschner A Kuhn J. [Avoidable mortality-a new indicator version for prevention reporting]. Bundesgesundheitsbl. 2022;65(1):116-25. Epub 20211208. doi: 10.1007/s00103-021-03458-y. 10.1007/s00103-021-03458-y PMC8732977 34878567

